# Electrophysiological changes in the conducting properties of fast pathway following modification of the slow pathway of the atrio ventricular node for atrio ventricular nodal re-entrant tachycardia

**DOI:** 10.12669/pjms.35.5.473

**Published:** 2019

**Authors:** Imran Khan, Bakhtawar Shah

**Affiliations:** 1 Dr. Imran Khan, FCPS. Department of Cardiology, Hayatabad Medical Complex, Peshawar, Pakistan; 2 Dr. Bakhtawar Shah, FCPS. Department of Cardiology, Hayatabad Medical Complex, Peshawar, Pakistan

**Keywords:** AVNRT, Electrophysiological changes, Non inducibility, Slow Pathway modification

## Abstract

**Objectives:**

To determine the possible changes in the conducting properties of the fast pathway after modification of the atrioventricular slow pathway for AVNRT which leads to the failure of the induction of tachycardia.

**Methods:**

This study was conducted in the Cardiac electrophysiology Laboratory of Hayatabad Medical Complex, Peshawar, Pakistan from March 2017 to March 2018. All the patients underwent radiofrequency modification of the slow pathway for AVNRT. Patients in whom typical AVNRT was inducible with demonstration of dual AV nodal physiology were included in the study.

**Results:**

A total of 171 cases were included in the study, 42 (25%) were males, mean age recorded was 47 ± 15 years. There were no significant changes pre and post ablation in the base line parameters like VV interval, atrioventricular nodal (AV nodal) Wenckebach cycle length, slow pathway effective refractory period (SPERP) or fast and slow pathways maximal Atrio His interval. However significant change was observed in the effective refractory period of the fast pathway 350±49 Vs 290±32 (p value 0.0001). The difference between slow and Fast pathway ERP was also decreased significantly 82±36 Vs 56± 24 (p value 0.004).

**Conclusion:**

Our study showed that ablation of AV nodal slow pathway for atrioventricular nodal reentrant tachycardia leads to changes in the effective refractory period of the fast pathway.

## INTRODUCTION

The most effective treatment of atrioventricular nodal re-entrant tachycardia (AVNRT) is modification of the slow pathway using radiofrequency ablation.^1^-^3^ Slow pathway is modified at the base of the Kock’s triangle. Modification of slow pathway leads to non inducibility of the tachycardia. Various mechanisms have been hypothesized for the non inducibility of AVNRT. The fast pathway effective refractory period (FPERP) is shortened after modification of the slow pathway. This change in the FPERP may be the cause of non inducibility of AVNRT. Other possible mechanisms may be changes in autonomic nervous system and thermal energy.^4^-^8^ Slow pathway modification may have an effect on the neuronal ganglionic plexus. The ganglionic plexus is anatomically located close to the coronary sinus ostium on the epicardial side. We ablated the slow pathway close to this anatomic location. Ablation in this location inhibits vagal input to the atrioventricular node (AVN) as well as in the atria.^9^,^10^ Also ablation of the slow pathway leads to an increase in the threshold to atrial fibrillation induction with atrial burst pacing suggesting local denervation of the parasympathetic nervous system as the possible mechanism.

Our objective was to determine the possible changes in the conducting properties of the fast pathway after modification of the atrioventricular slow pathway for AVNRT which leads to the failure of the induction of tachycardia.

## METHODS

This study was conducted in the Electrophysiology Laboratory of Hayatabad Medical Complex Peshawar Pakistan from March 2017 to August 2018. All patients who (1) underwent successful radiofrequency modification of the slow pathway for typical AVNRT, (2) age less than or equal to 15 years, (3) typical AVNRT that could be induced with atrio-His (AH) jump, (4) and typical AVNRT as the only diagnosis were included in the study. The sampling method was consecutive random.

Exclusion criteria included (1) no AH Jump with decremental atrial pacing, (2) non sustained AVNRT, even with isupril infusion, (3) inducible tachycardia after ablation with or without isoprinaline. Non inducibility of AVNRT after ablations was labelled as procedural success. (Proc Natl Acad Sci U S A)

All AV nodal blocking drugs were stopped for five half-lives pre-procedure. Procedure eligible patients received conscious sedation with midazolam and/or fentanyl. A detailed written informed consent form describing the electrophysiologic study, procedure of radiofrequency ablation and the possible complications of the procedure was obtained from the patient.

Three quadripolar electrode catheters and one decapolar catheter were introduced. Four wires study was done. Three quadripolar (St. Jude Medical Inc. USA) were placed in the high right atrium, at the site of His bundle and RV apex introduced via left and right femoral veins. The decapolar catheter (dynamic tip steerable diagnostic catheter Boston Scientific) was introduced via the left femoral vein (6-Fr sheath) and placed in the coronary sinus; ablation catheter was introduced via 7-Fr sheath in the right femoral vein. Atrioventricular and ventriculoatrial block was documented.

AVNRT was induced using atrial extra stimulus with Bloom Stimulator (Fisher medical technologies, USA) with or without isoprinaline. Electrophysiological parameters pre and post procedure were measured. Baseline ECG intervals and intracardiac electrograms were measured. Baseline parameters including the VV interval, AV node Wenckebach cycle length (AVN-WCL), fast and slow pathways ERP and their difference, and the maximal fast and slow pathways atrio-His interval were measured. Electrographic and a fluoroscopic guided RF ablation was applied at the base of the kock’s triangle. If slow junctional rhythm was not achieved within the first 10-20 seconds of ablation or if the impedance rise was too high, RF energy was stopped. The RF power and temperature was kept at 40 and 60 degrees respectively. Once slow junctional rhythm was achieved, radiofrequency ablation was continued for one minute. Successful modification was defined as good junctional rhythm during slow pathway modification, non inducibility of AVNRT and the inability to document two or more AVN echoes. If the patient initially needed isoprinaline for induction, isoprinaline was given for tachycardia induction. Post procedure determination of AH interval, Fast pathway and slow pathway difference (FP-SP) in ERP, AVN Wenckebach cycle length (WBCL), and anterograde and retrograde block of the AVN was documented. To determine reproducibility, FPERP and SPERP were measured twice. Descriptive statistics (frequency, percentages and mean ± SD) were used for quantitative variables. Data was analyzed by SPSS version 23. Chi square test was used for categorical variables and student-t test for numerical variables. P-value of 0.05 was taken as significant. All results were presented in the form of tables.

**Fig.1:**
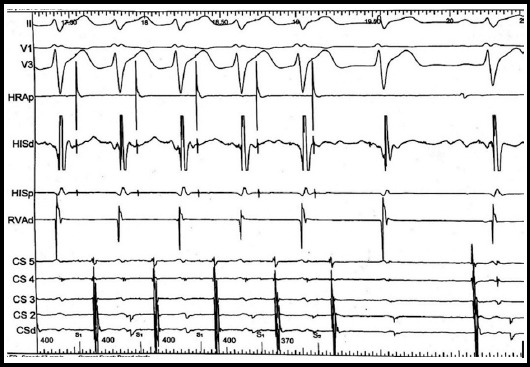
Representative Case showing atrioventricular node parameters. The ERP of the fast pathway, defined as the longest premature coupling that fails to conduct along the fast pathway. ERP, effective refractory period

**Fig.2:**
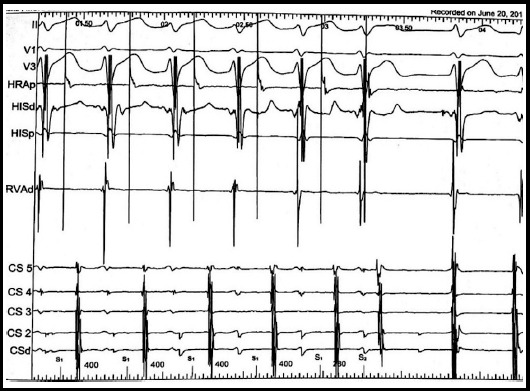
Representative case showing Fast Pathway effective refractory period (ERP) after slow pathway modification for typical AVNRT.

## RESULTS

Of the 200 cases included in the study 29 were excluded according to the inclusion and exclusion criteria: 42 (25%) were male, mean age 47±15. All patients underwent successful RF ablation, AVNRT was thus non inducible. There were no statistically significant changes pre and post ablation in the VV interval, AV nodal Wenckebach cycle length (AVN-WCL), VA and AV block, slow pathway effective refractory period (SPERP), maximal AtrioHis (AH) intervals of the fast and slow pathway. The electrophysiologic dimensions pre and post ablation are shown in Table-I.

**Table I:** Electrophysiologic parameters pre and post Radiofrequency Ablation (RFA).

Electrophysiologic Parameters (msec)	Before RFA	After RFA	P value
VV interval)	588±155	561±139	0.06
AVN-Wenchbach CL	342±50	328±27	0.30
Fast pathway Effective RP	354±55	298±66	0.001
Slow pathway Effective RP	244±48	245±48	0.60
SPERP-FPERP	92±39	55±33	0.007
Fast pathway maximal AH interval	161±45	173±42	0.40
Slow pathway maximal AH interval.	324±90	342±118	0.60
Maximal AH SP-FP	150±66	136±101	0.5
AtrioVentricular block	371±42	340±56	0.80
Ventriculoatrial block	365±59	378±61	0.55

The slow pathway electrophysiological properties did not change significantly after RF ablation. Baseline VV interval was 588±155msec and AVN-WCL was 342±50 msec. Changes in the VV interval (p=0.06) or Wenckebach cycle length AV node (p=0.3) were non-significant pre and post RF ablation. The fast pathway ERP at baseline was 354±55msec, and was shortened to (298±66, p<0.001) post procedure. However, slow pathway ERP did not change significantly pre and post ablation (p=0.07). The fast and slow pathways ERPs difference significantly decreased (p=0.007). There was no significant change in the fast (p=0.4) and slow pathway (p=0.60) maximal AH interval and their differences (p=0.5) as shown in Table-II.

**Table II:** Demographic and Clinical Characteristics.

Age, years	47±15.
Male, n (%)	42(25%)
Duration of tachycardia, months	8.8 ± 7.1
LVEDd, mm	45±13.1
Ejection fraction (%)	63.8±4.8
LA, mm	35.2 ±1.5
No. of ablations	5.1±2.5
Ablation time (sec)	160.3±12.3
Flouro Time(sec)	300±40.2
Total procedure time, min	12±4.1

## DISCUSSION

Cardiac arrhythmias including AVNRT can be safely and effectively ablated with radiofrequency. The effect of RF ablation on the ERP of the fast pathway immediately after slow pathway ablation has been demonstrated in many studies.^8^-^16^ Our study showed that the electrophysiological properties of the fast pathway change after slow pathway modification. Complete elimination of the slow pathway is not mandatory for the radiofrequency ablation as a treatment of AVNRT, rather the slow pathway is modified in 24 - 68% of the patients.^17^-^19^ The tachycardia is non inducible despite the failure to completely eliminate the slow pathway. This implies that change in the electrophysiological properties of the AVN following the ablation of the slow pathway may be reasons for the non inducibility of the tachycardia.

Our study demonstrated that modification of the slow pathway for AVNRT cause a decrease in the slow pathway ERP and thus the conduction through AVN is improved. Similar to our study, another study showed a significant reduction in the fast pathway ERP with no effect on the slow pathway ERP as well as no effect on the AH interval of the fast and slow pathway post modification of the slow pathway.^20^ Contrary to our study, a study showed decrease in the maximal AH interval of slow pathway with no significant changes in the differences of other parameters.^21^ Lastly, Posan et al. demonstrated an increase in slow pathway ERP and thus a decrease of differences between the ERP of the fast and slow pathways.^22^ This was described as the possible mechanism of the non inducilbity of AVNRT.

Radiofrequency energy cause tissue injury and thus the slow pathway is unable to sustain the arrythmia.^23^ A decrease in post ablation slow pathway AH interval leads to paradoxically enhanced conduction along the slow pathway. This also cause decrease in the ERP of the fast pathway which is the possible mechanism of single AVN echo.

Atrial tissue especially perinodal tissue and the transitional cells in the reentry circuit is thought to be involved in the mechanism of AVNRT.^7^,^12^,^18^ Histologically and morphologically the AVN has not been proved to have changed after slow pathway modification.

Various hypothesis have been suggested for these changes in electrophysiological properties of the AVN after slow pathway ablation. Studies have shown that ablation of the slow pathway at the base of Koch’s triangle may be responsible for the autonomic changes in the ganglionic plexus. The extrinsic vagal fibers integrate at the ganglionic plexus at the base of kock’s triangle. Both the right and left vagus nerves give autonomic branches which pass through the ganglionic plexus to the AVN.^24^,^25^ Just behind the endocardially located coronary sinus ostium the ganglionic plexus is anatomically located. Vagal activity to the AVN is inhibited after ablation in this area.^25^ Also slow pathway ablation for AVNRT increases the threshold to pacing-induced atrial fibrillation, suggesting local vagal (parasympathetic) denervation as the possible mechanism.

Therefore, ablation at the base of Koch’s triangle for the AV nodal slow pathway may cause denervation of vagus nerve and thus non inducibility of AVNRT. Our study is basically based on the assumption that the electrical properties of the AVN especially fast pathway changes after sow pathway modification. The modification of slow pathway leads to changes in the AVN such that communication between the slow and fast pathway is disrupted, causing AVNRT to be non-inducible. The autonomic changes following modification of the slow pathway may be the possible mechanism for the non inducibility of AVNRT. Our study documented a decrease in the fast pathway ERP but we could not document the actual reason for the decrease in the fast pathway ERP. Changes in the autonomic ganglionic plexus after slow pathway ablation have been attributed to the changes in the electrophysiological properties of the AVN. Yin et al.^25^ have demonstrated changes in the autonomic nervous system as the possible cause for atrial ERP and AVN ERP and thus non inducibility of ERP. This may be the limitation of our study.

## CONCLUSION

Our study conclude that ablation of the slow pathway of atrioventricular node for AVNRT leads to changes in the effective refractory period and thus improved conduction along the fast pathway. Although dual AV nodal physiology can still be documented in most of these patients, tachycardia cannot be initiated. This may be the possible mechanism of non inducibility of the tachycardia.

### Author’s Contribution

**IK:** conceived, designed and did statistical analysis & editing of manuscript.

**IK:** BS did data collection and manuscript writing.

**IK:** did review and final approval of manuscript.
